# Enhanced cholera surveillance to improve vaccination campaign efficiency

**DOI:** 10.1038/s41591-024-02852-8

**Published:** 2024-03-05

**Authors:** Hanmeng Xu, Kaiyue Zou, Juan Dent, Kirsten E. Wiens, Espoir Bwenge Malembaka, Godfrey Bwire, Placide Welo Okitayemba, Lee M. Hampton, Andrew S. Azman, Elizabeth C. Lee

**Affiliations:** 1 Department of Epidemiology, Johns Hopkins Bloomberg School of Public Health, Baltimore, MD USA; 2 Department of Epidemiology and Biostatistics, College of Public Health, Temple University, Philadelphia, PA USA; 3 Center for Tropical Diseases and Global Health, Université Catholique de Bukavu, Bukavu, Democratic Republic of the Congo; 4 Division of Public Health Emergency Preparedness and Response, Ministry of Health, Kampala, Uganda; 5 Makerere University School of Public Health, Kampala, Uganda; 6 Programme National d’Elimination de Choléra et lutte contre les autres Maladies Diarrhéiques, Kinshasa, Democratic Republic of the Congo; 7 Gavi, the Vaccine Alliance, Geneva, Switzerland; 8 Geneva Centre for Emerging Viral Diseases, Geneva University Hospitals, Geneva, Switzerland; 9 Division of Tropical and Humanitarian Medicine, Geneva University Hospitals, Geneva, Switzerland

**Keywords:** Bacterial infection, Computational models

## Abstract

Systematic testing for *Vibrio cholerae* O1 is rare, which means that the world’s limited supply of oral cholera vaccines (OCVs) may not be delivered to areas with the highest true cholera burden. Here we used a phenomenological model with subnational geographic targeting and fine-scale vaccine effects to model how expanding *V. cholerae* testing affected impact and cost-effectiveness for preventive vaccination campaigns across different bacteriological confirmation and vaccine targeting assumptions in 35 African countries. Systematic testing followed by OCV targeting based on confirmed cholera yielded higher efficiency and cost-effectiveness and slightly fewer averted cases than status quo scenarios targeting suspected cholera. Targeting vaccine to populations with an annual incidence rate greater than 10 per 10,000, the testing scenario averted 10.8 (95% prediction interval (PI) 9.4–12.6) cases per 1,000 fully vaccinated persons while the status quo scenario averted 6.9 (95% PI 6.0–7.8) cases per 1,000 fully vaccinated persons. In the testing scenario, testing costs increased by US$31 (95% PI 25–39) while vaccination costs reduced by US$248 (95% PI 176–326) per averted case compared to the status quo. Introduction of systematic testing into cholera surveillance could improve efficiency and reach of global OCV supply for preventive vaccination.

## Abstract (web summary)

A modeling study of 35 cholera-affected countries in Africa showed that introducing cholera surveillance could improve the effectiveness and cost-effectiveness of preventive cholera vaccination campaigns.

## Main

Cholera remains a major public health threat in areas with limited access to safe water and sanitation services. Africa bears a substantial part of the global burden of cholera with an estimated 87 million people living in high-incidence districts (that is, mean annual incidence rate >1 suspected case per 1,000 people)^1,2^. However, these estimates rely primarily on data from passive clinical surveillance with infrequent laboratory confirmation and may not reflect true cholera burden.

Cholera incidence varies greatly across space and time. The majority of suspected cases reported in Africa during 2010–2016 were from less than 5% of the population^1,3^, and 65% of reported outbreaks during 2010–2019 occurred in only four countries^4^. Even in high-incidence populations, cholera transmission can span the endemic–epidemic continuum, including locations with year-round transmission and locations with outbreaks recurring every three to five years and no reported cases in interim periods^5^. This heterogeneity challenges national surveillance systems, which may need multiple case definitions and reporting protocols to accommodate different transmission settings. When both cholera epidemiology and surveillance reporting vary widely, targeting disease control measures efficiently can be extremely difficult.

Previous work has shown that targeting cholera control to areas with high historical burden can make substantial improvements to the cost-effectiveness and public health impact of these interventions^6^. Geographic targeting is critical for the rollout of disease-specific, planned control measures such as preventive vaccination campaigns; and only 33 million doses were shipped out of 72 million doses requested in 2022 (ref. ^7^). Yet the cholera surveillance programs required to enable such targeting are lacking. While most cholera-affected countries in Africa perform passive clinic-based cholera surveillance, there is substantial variation in case definitions, reporting coverage, data quality and case detection practices^3,8–10^. Further, systematic laboratory confirmation of suspected cholera cases through culture and polymerase chain reaction (PCR) testing is challenging due to limited laboratory resources and supply chains. Among suspected cholera outbreaks in Africa from 2010 to 2019, laboratory testing data were reported in 25% of outbreaks and only 13% reported at least one confirmed cholera case^4^. While rapid diagnostic tests (RDTs) for *Vibrio cholerae* O1/O139 detection are being increasingly adopted for outbreak detection and case screening, their widespread use is relatively new, performance across tests and in different settings is variable, and global standards for their use and interpretation for surveillance are still in their infancy^8,11^.

A previous systematic review and meta-analysis estimated that an average 52% of suspected cholera cases were true cholera, but this proportion varied widely across space and time with a range of 0.01% to 100% (ref. ^12^). With suspected cases coming from other diarrhea-causing pathogens such as enterotoxigenic *Escherichia coli*, *Cryptosporidium* and *Shigella*^13–15^, the current practice of prioritizing the world’s limited supply of oral cholera vaccine (OCV) using primarily suspected cholera surveillance could be highly inefficient. Fine-scale OCV targeting supported by improved bacteriological confirmation capacity would substantially increase campaign efficiency and vaccine impact while simultaneously reducing the number of campaign sites and target population sizes.

In this Article, focusing on 35 cholera-affected countries in Africa where district-level (subnational) cholera incidence estimates are available, we build upon an existing strategic modeling framework^6^ to explore the potential gains in preventive vaccination campaign impact and efficiency that may be observed with improved *V. cholerae* O1/O139 confirmation capacity (Table 1). We modeled the impact of vaccination campaigns under different scenarios and calculated the vaccine impact, vaccination campaign efficiency and cost-effectiveness relative to a scenario with no vaccination.

**Table 1:** Policy summary table

Background	Systematic laboratory confirmation of suspected cholera cases is rare, which means the world’s limited supply of OCV may be delivered to geographic areas that do not have the highest true burden of *V. cholerae*.
Main findings and limitations	Our strategic modeling study in cholera-affected regions of Africa found that targeting vaccines on the basis of the burden of systematically confirmed cholera resulted in more averted cases per vaccine used, higher cost-effectiveness when accounting for the costs of testing and vaccine delivery, with a relatively small reduction in the absolute magnitude of averted cases across model settings. Limitations of this study include not accounting for annual variability in cholera burden and immunity and simplified assumptions of testing and vaccine targeting compared to reality. Due to the model assumptions, results should be interpreted relative to the other reported scenarios and not as absolute projections.
Policy implications	Introduction of systematic testing into cholera surveillance could improve efficiency and reach of global OCV supply for preventive vaccination. Future investigation should consider what drives country-level variability in the optimal systematic testing strategy to inform specific surveillance system designs.

## Results

### Impact of testing on OCV targeting and efficiency

**Fig. 1:**
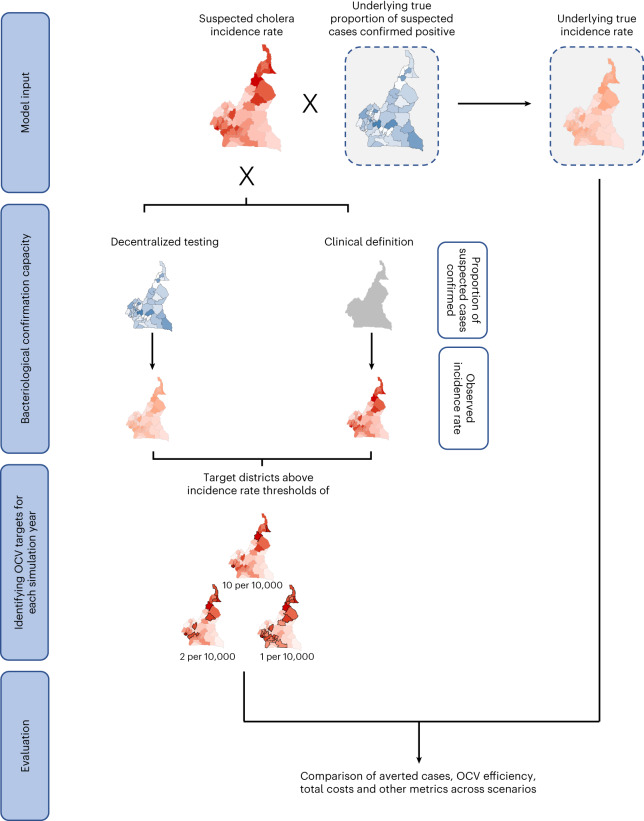
Conceptual depiction of relationship between model inputs and modeling scenarios. Model inputs include suspected cholera incidence rate maps and an underlying true positivity, from which a true *V. cholerae* incidence rate map is derived. To determine how OCV is targeted, a bacteriological confirmation capacity setting is applied. Under the decentralized testing setting, true positivity is assumed to be known at the district level, and the true incidence rate map is observed. Under the clinical definition setting, only suspected cholera incidence is observed. Districts are targeted for OCV in a simulation year if the mean observed incidence rate over the past 5 years exceeds one of three thresholds, 10 per 10,000, 2 per 10,000 or 1 per 10,000 population, and the location has not been vaccinated in the last 3 years. Models are simulated and public health impact and cost-effectiveness are evaluated with true averted cases, true averted cases per 1,000 FVPs (OCV efficiency) and total costs of testing and OCV campaigns, among other metrics.

Scenarios that targeted OCV campaigns using surveillance with enhanced bacteriological confirmation capacity through systematic testing of suspected cholera cases with RDT and culture (‘decentralized testing’ and ‘centralized testing’ scenarios) always had higher OCV efficiency than those that targeted campaigns using only suspected cholera case definitions (‘clinical definition’ scenario) (Fig. 1, Extended Data Tables 1 and 2). When districts (subnational units within countries) with an observed cholera incidence rate over 10 per 10,000 population were targeted with OCV, the decentralized testing and clinical definition scenarios fully vaccinated 30.1 (95% prediction interval (PI) 24.4–37.9) and 65.2 (95% PI 59.8–71.0) million individuals and averted 0.33 (95% PI 0.26–0.41) and 0.45 (95% PI 0.38–0.53) million cases, thus yielding an OCV efficiency of 10.8 (95% PI 9.4–12.6) and 6.9 (95% PI 6.0–7.8) averted cases per 1,000 fully vaccinated persons (FVPs), respectively (Table 2). This represented 19.2% (95% PI 16.7–22.3) of true cases averted in scenarios with systematic decentralized testing and 26.5% (95% PI 24.4–28.5) in those without.

**Table 2:** FVPs, averted cholera cases, OCV campaign efficiency and percent of true cases averted across different modeling scenarios, 2022–2035

Threshold	Fully vaccinated population (million)		Averted cases (million)		OCV efficiency (averted cases per 1,000 FVPs)		Percent of true cases averted	
	Clinical definition	Decentralized testing	Clinical definition	Decentralized testing	Clinical definition	Decentralized testing	Clinical definition	Decentralized testing
1/10,000 per year	391.8 (378.8–412.2)	274.6 (261.1–285.8)	0.76 (0.69–0.84)	0.71 (0.64–0.8)	1.9 (1.7–2.2)	2.6 (2.4–2.9)	44.7 (44.3–45.3)	41.8 (41–42.9)
2/10,000 per year	275.2 (264.5–283.9)	165.2 (153.9–179.4)	0.7 (0.64–0.79)	0.62 (0.55–0.71)	2.6 (2.3–2.8)	3.7 (3.4–4.2)	41.5 (40.8–42.7)	36.5 (35.4–38.1)
10/10,000 per year	65.2 (59.8–71)	30.1 (24.4–37.9)	0.45 (0.38–0.53)	0.33 (0.26–0.41)	6.9 (6–7.8)	10.8 (9.4–12.6)	26.5 (24.4–28.5)	19.2 (16.7–22.3)

For combinations of modeling scenarios that vary by incidence rate threshold and bacteriological confirmation capacity, we report the median estimates and 95% PIs in parentheses for FVP, true averted cases (millions), true averted cases per 1,000 FVPs (OCV efficiency) and percentage of true cases averted. ‘Clinical definition’ refers to a scenario without testing of suspected cases, while ‘decentralized testing’ refers to a scenario with systematic testing of suspected cases with RDT and culture in district-level laboratories.

**Extended Data Fig. 1:**
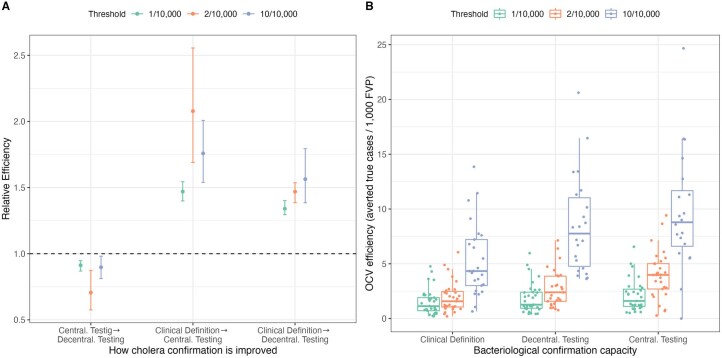
OCV campaign efficiency by cholera bacteriological confirmation capacity and vaccination threshold for district-level targeting scenarios. (A) Relative OCV campaign efficiency under different bacteriological confirmation capacity improvements for district-level targeting scenarios. The point represents the median relative efficiency across simulations (n = 200 simulations), and the vertical lines show 95% prediction intervals. The horizontal dashed line represents ‘No improvement of OCV efficiency’ (that is, relative efficiency = 1). (B) This figure shows the distribution of median OCV campaign efficiency by country. Each point represents the median estimate across simulations for one country (n = 35 countries), and the boxplot shows the distribution of the country medians.

Across targeting thresholds of 1, 2 and 10 cases per 10,000 population, the median of the clinical definition scenarios vaccinated 1.43, 1.67, and 2.17 times more people than the median of the decentralized testing scenarios, while averting 1.07, 1.13, and 1.37 times as many true cholera cases at the median, respectively. Thus, OCV efficiency of decentralized testing scenarios was 1.34 (95% PI 1.29–1.40), 1.47 (95% PI 1.39–1.54) and 1.56 (95% PI 1.39–1.79) times higher than clinical definition scenarios for the three targeting thresholds, respectively (Extended Data Fig. 1).

Scenarios with enhanced confirmation capacity led to a more focused delivery of vaccines to people living in high-incidence-rate areas. For example, 97.8% (95% PI 92.8–100%) of FVP lived in areas where the true incidence rate exceeded 10 per 10,000 population in the relevant decentralized testing scenario, in contrast with 43.1% (95% PI 35.2–51.8%) in the analogous clinical definition scenario (Extended Data Table 2). When targeting areas with an incidence rate above 10 per 10,000, introducing decentralized testing reduced the total number of districts targeted by the OCV campaign from 272 (95% PI 238–319) to 130 (95% PI 107–171), and reduced the unique number of districts targeted from 119 (95% PI 105–137) to 60 (95% PI 48–76) (Extended Data Table 2).

### Impact of testing on OCV cost-effectiveness

**Extended Data Fig. 2:**
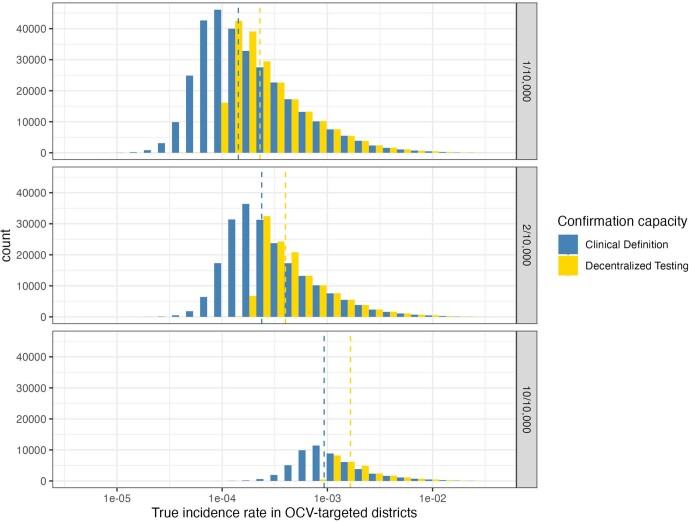
Baseline true incidence rate of targeted districts. The histograms show the distributions of true incidence rate of districts targeted by OCV campaigns across 2022-2030 for the three targeting incidence rate thresholds. The incidence rate value is the true incidence rate at the year of being targeted. The steel blue bars represent the distribution of the ‘Clinical Definition’ confirmation scenario, and the gold bars represent the distribution of the ‘Decentralized Testing’ scenario. The incidence rate (x-axis) is displayed on the log scale. The dashed lines mark the medians of the distributions.

We found that systematic decentralized testing greatly reduced the combined cost of testing and OCV campaigns across targeting thresholds compared to the clinical definition scenario. For example, for the targeting threshold of 1 per 10,000, testing reduced the total cost per averted case from US$2,452 (95% PI 2,153–2,707) to US$1,829 (95% PI 1,653–2,017) (Table 3). Each US dollar spent on testing suspected cholera cases reduced OCV campaign costs by US$61 (95% PI 52–69). Decentralized testing also increased OCV efficiency from 1.9 (95% PI 1.7–2.2) to 2.6 (95% PI 2.4–2.9) averted cases per 1,000 FVPs but averted a slightly lower percent of true cases—41.8% (95% PI 41.0–42.9) versus 44.7% (95% PI 44.3–45.3) in the clinical definition scenario (Table 2). Decentralized testing scenario outcomes were not equivalent to clinical definition scenarios with higher vaccine targeting threshold, as systematic testing had the added effect of reducing variability in the detection and targeting of high-incidence-rate areas (Extended Data Fig. 2).

**Table 3:** Cost-effectiveness of introducing decentralized testing of suspected cholera cases, 2022–2035

Threshold	Total cost per averted case (US$)		OCV cost reduction per averted case (US$)	Test cost per averted case (US$)	OCV cost reduction per test dollar spent (US$)
	Clinical definition	Decentralized testing			
1/10,000 per year	2,452 (2,153–2,707)	1,829 (1,653–2,017)	631 (509–731)	10 (9–11)	61 (52–69)
2/10,000 per year	1,841 (1,658–2,042)	1,272 (1,142–1,413)	587 (473–682)	13 (12–14)	45 (39–51)
10/10,000 per year	685 (603–780)	468 (405–543)	248 (176–326)	31 (25–39)	8 (6–9)

We report the median estimates and 95% PIs for total costs per averted cases and metrics that demonstrate tradeoffs in testing and OCV costs between clinical definition and decentralized testing settings. OCV cost reduction (due to systematic decentralized testing) per averted case is the difference between OCV cost per averted case in the decentralized testing and clinical definition scenarios. Test cost per averted case is the cost of testing in decentralized testing scenarios; no tests were performed in clinical definition scenarios. OCV cost reduction per test dollar spent is the ratio of OCV cost reduction per averted case and test cost per averted case.

### Heterogeneity in testing impact across countries

**Fig. 2:**
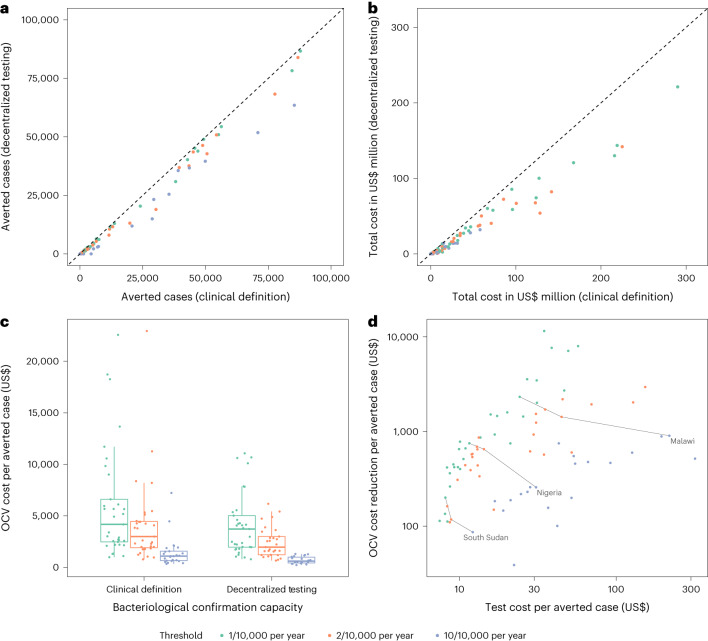
Country-level cost and cost-effectiveness by introducing decentralized testing of suspected cholera cases, 2022–2035. **a**, Comparison of averted true cholera cases between ‘clinical definition’ and ‘decentralized testing’ scenarios. Each point represents a modeled country median across simulations; the *x* axis and *y* axis are the number of true averted cases in the clinical definition and decentralized testing scenarios and the dashed line shows where *y* = *x*. **b**, Comparison of median country total cost (sum of OCV campaign cost and testing cost) between clinical definition and decentralized testing scenarios. **c**, Distribution of median cost of OCV campaign per averted true cholera case by country, under district-level OCV targeting setting. Each point represents the median estimate for one individual modeled country, and the boxplots show the distributions of the country medians (*n* = 35 countries), where the box demarcates the 25th, 50th and 75th percentile and the whiskers extend beyond the 25th and 75th percentiles by 1.5 times the interquartile range. **d**, Cost tradeoff between decentralized bacteriological confirmation of *V. cholerae* by RDT or culture and OCV campaign, under district-level OCV targeting setting. Each point represents an individual county. The *x* axis and *y* axis are the median cost spent per averted case and the median cost reduction per averted case, for a specific country. The gray lines link the data points of the three targeting thresholds for three selected countries, South Sudan, Nigeria and Malawi.

**Extended Data Fig. 3:**
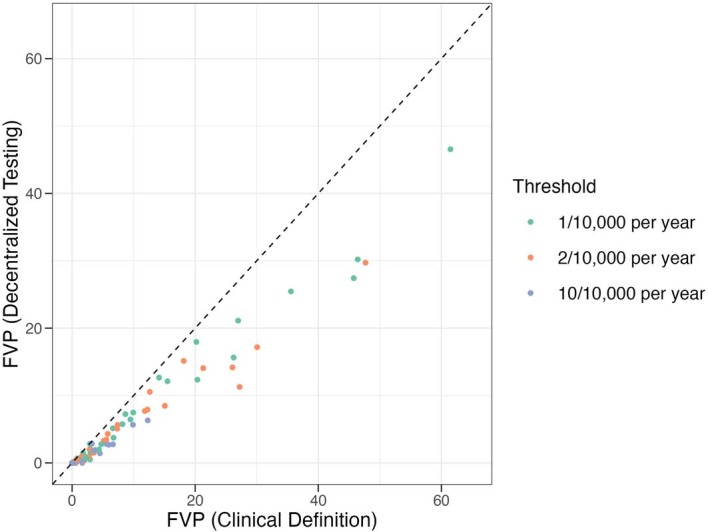
Country-level fully vaccinated persons (FVP) by introducing ‘Decentralized Testing’ of clinical cholera cases, 2022-2035. Comparison of fully vaccinated persons (FVP) between ‘Clinical Definition’ and ‘Decentralized Testing’ scenarios, under district-level OCV targeting setting. Each point represents an individual modeled country; the x-axis and y-axis are respectively the median FVPs of the ‘Clinical Definition’ and ‘Decentralized Testing’ scenarios for one specific country; the dashed line marks where y = x.

Across country-level outputs, scenarios that introduced decentralized testing into surveillance systems averted slightly fewer true cholera cases than those using a suspected case definition to target OCV (Fig. 2a), while vaccinating many fewer people (Extended Data Fig. 3). Consequently, decentralized testing scenarios achieved a higher OCV campaign efficiency (Table 2) with lower total costs than comparable clinical definition scenarios (Fig. 2b). In addition, decentralized testing scenarios led to more cost-effective OCV campaigns and reduced across-country heterogeneity in cost-effectiveness (as measured by OCV cost per case averted) as compared to clinical definition scenarios (Fig. 2c).

**Extended Data Fig. 4:**
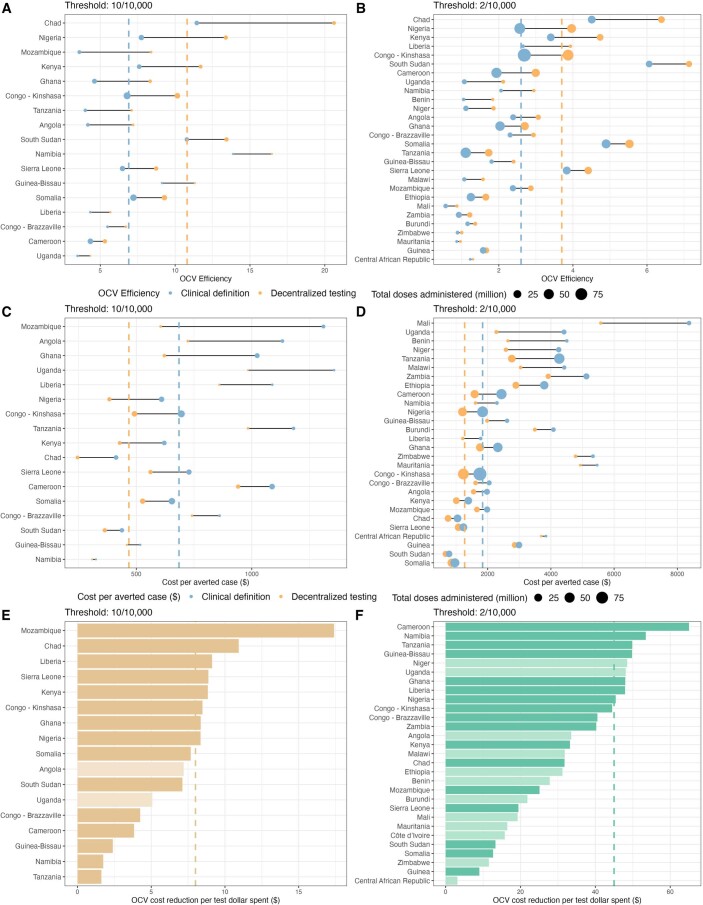
Total doses administered, total cost per averted cases, and OCV cost reduction per test dollar spent compared between clinical definition testing and decentralized testing scenarios by country. Country-level OCV efficiency for the clinical definition and decentralized testing settings are depicted for the (A) 10 per 10,000 and (B) 2 per 10,000 incidence rate thresholds. Country-level costs per averted case for the clinical definition and decentralized testing settings are depicted for the (C) 10 per 10,000 and (D) 2 per 10,000 incidence rate thresholds. For panels (A)-(D), clinical definition results are in blue, decentralized testing results are in goldenrod, and point sizes refer to the total doses administered in a given scenario (in millions). Country-level OCV cost reductions per test dollar spent are depicted for the (E) 10 per 10,000 and (F) 2 per 10,000 incidence rate thresholds. For all panels, the vertical dashed lines represent the cross-continent median estimate for a given setting (matched by color). A baseline incidence rate for each country was calculated by taking the mean of the population-weighted gridded 2010-2016 mean annual suspected cholera incidence rate across 1000 posterior samples^1^. For panels (E) and (F), countries with a 2010-2016 mean annual incidence rate greater than or equal to the median of the country baseline incidence rates (9.1*10^−5^ per year) had dark-shaded bars while countries below the median had light-shaded bars.

Our results suggest that, when OCV use expands to include moderate incidence settings, testing becomes more critical to OCV campaign cost reduction (Fig. 2d). For example, when we lowered the targeting threshold from 10 per 10,000 to 1 per 10,000 in Nigeria, test costs per true averted case declined from US$31 to US$12 while the reduction in OCV costs per averted case increased from US$353 to US$1,806 (Fig. 2d). Similarly, in South Sudan and Malawi, the test cost per true averted case declined from US$12 to US$8 and from US$218 to US$24, while the OCV cost per averted case increased from US$351 to US$790 and from US$1,029 to US$4,283, respectively. The improvement in OCV efficiency and cost per averted case with the introduction of testing varied widely across countries, and it did not appear that high-burden countries would universally experience larger OCV cost reductions per test dollar spent (Extended Data Fig. 4).

### Comparison of decentralized and centralized testing

**Fig. 3:**
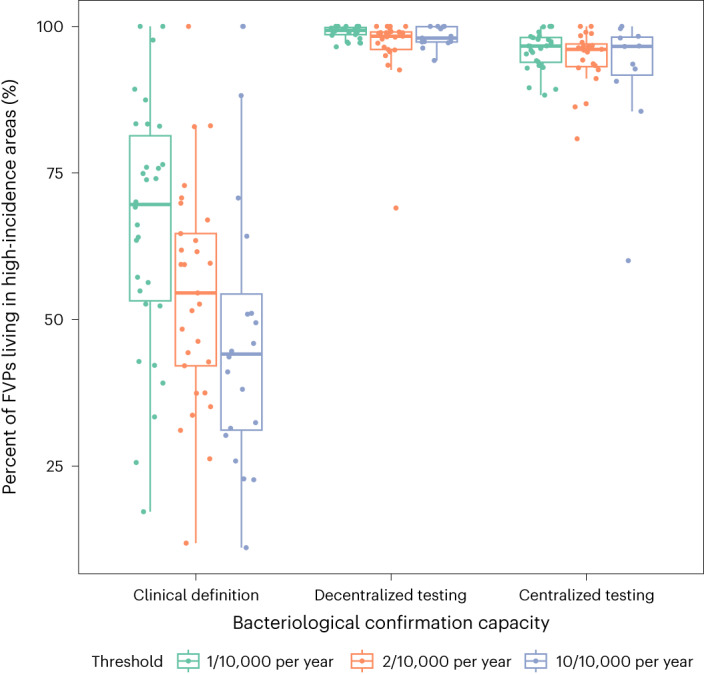
Percent of FVPs living in high-incidence-rate administrative units by country. For each modeling scenario, each point represents the country median of percent of FVP living in administrative units with a true incidence rate exceeding the incidence rate threshold indicated by the color. The boxplot represents the distribution of country-level medians (*n* = 35 countries), where the box demarcates the 25th, 50th and 75th percentile and the whiskers extend beyond the 25th and 75th percentiles by 1.5 times the interquartile range.

**Extended Data Fig. 5:**
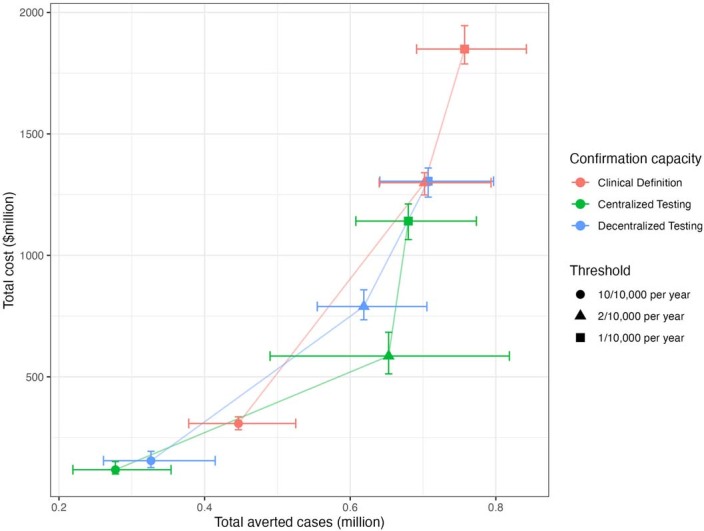
Tradeoffs between total OCV and testing costs and total averted cases by model scenario (across all countries and model simulation years). The point represents the median and the horizontal and vertical bars represent the 95% prediction intervals of the two metrics (n = 200 simulations). Lines connect scenario projection points for the same confirmation capacity setting.

We also compared systematic decentralized and centralized testing strategies. Centralized testing scenarios had slightly higher OCV efficiency and targeted slightly fewer people and administrative units than decentralized testing scenarios (Extended Data Table 2). For example, when targeting districts with an observed incidence rate above 10 per 10,000, the centralized testing scenario fully vaccinated 22.8 (95% PI 19.3–29.5) million individuals, averted 0.28 (95% PI 0.22–0.35) million cases and averted 12 (95% PI 10.3–14.4) cases per 1,000 FVPs (Extended Data Table 2).This represented a more focused campaign with fewer averted cases but greater efficiency than the decentralized testing results reported above, though the 95% PIs overlapped (Table 2). When considering tradeoffs between total cost and averted cases, the centralized testing setting averted more cases for lower total costs than the decentralized testing setting when targeting districts above the 2 per 10,000 threshold (Extended Data Fig. 5). However, the higher costs of decentralized testing yielded more averted cases when the vaccination targeting thresholds were at 1 per 10,000 or 10 per 10,000. The proportion of FVP living in truly high-incidence-rate areas was lower in the centralized testing scenario, with 94.3% (95% PI 83–99%) in the scenario targeting districts above the threshold of 10 per 10,000 (Fig. 3 and Extended Data Table 2).

## Discussion

Our study builds on previous OCV targeting work^6^ by proposing how specific surveillance improvements—that is, systematic confirmatory testing of suspected cholera cases—could improve the targeting efficiency of preventive campaigns and extend the reach of the limited global supply of OCVs^16^. When districts with an observed mean annual incidence rate over 10 per 10,000 were targeted for vaccination, the introduction of systematic decentralized testing increased OCV campaign efficiency by about 60%, used nearly 70 million fewer doses in a 9-year period, and reduced vaccination costs by US$8 for every US dollar spent on testing, while observing a 25% decrease in true cases averted. In scenarios where OCV use was more expansive (that is, a lower incidence rate threshold was used to target districts for OCV), the introduction of testing produced smaller gains in OCV campaign efficiency but reduced the number of doses used and the amount spent on testing. This suggests that testing will become even more critical for OCV targeting as preventive OCV use becomes more common in lower-burden settings.

Our scenarios compared two systematic testing strategies that were based on recent Global Task Force on Cholera Control (GTFCC) guidance on public health surveillance in areas with confirmed outbreaks^17^. Compared to the centralized testing scenario, which was defined as systematic testing with culture in a single reference laboratory, decentralized testing used a combination of RDT and culture that resulted in a greater total number of tests performed and greater accuracy in the estimated *V. cholerae* positivity. The centralized testing scenario had the unanticipated effect of slightly increasing OCV efficiency, as only the highest-incidence-rate districts remained above the OCV targeting threshold after accounting for reductions in culture sensitivity due to transport and delays.

A robust laboratory surveillance system would most likely be a combination of the centralized and decentralized testing scenarios, with perhaps multiple national reference laboratories and smaller, dispersed laboratories with capacity to perform a mix of RDT and culture among suspected cases in their communities^18^. Such a system could gain the advantages from both approaches, with better quality control and standardization of testing in reference laboratories, and improved timeliness in outbreak confirmation from decentralized testing. As evidenced by the heterogeneity in testing impact across countries and variability in the optimal testing strategy at different vaccine targeting thresholds, enhanced testing strategies need to be adapted to the local context. High- and low-burden countries will necessarily have different approaches to cholera control, and the optimal testing strategy may vary based on the spatial distribution of burden, testing capacity and other specifics of the surveillance system. Future studies should consider how testing strategies may be optimally adapted to different burden, laboratory capacity and surveillance contexts, and how optimization may be sensitive to factors that also alter diagnostic test sensitivity in centralized and decentralized testing scenarios. These extensions could inform targeted guidance on the implementation of systematic testing in specific countries.

Our modeling scenarios do not capture notable challenges in confirmatory testing faced by many cholera-affected countries. We assumed that reporting and testing of cases would be spatially homogeneous within a district, although differences in access to care, health behavior, health care capacity and testing capacity could cause spatial heterogeneity. Even variation in antibiotic usage may bias reporting, as this has been observed to reduce culture sensitivity^19^. In addition, our cost-effectiveness analyses do not include the substantial resource, training and supply-chain difficulties of developing and maintaining a laboratory prepared to perform culture at any time.

While several cholera RDTs are now available and beginning to have wide usage, none of the products has received World Health Organization prequalification and only recently has GTFCC released guidance that integrates RDTs into public health cholera surveillance strategy^11,20,21^. RDT evaluation studies so far have found high variability in sensitivity and specificity across products and protocols concurrent with concerns about the accuracy of gold-standard culture results^11^, leading some to question the best uses of these tools. Nevertheless, use and confidence in cholera RDTs, supported by a comprehensive RDT evaluation with a standardized protocol, is critical for expanding the network of laboratories where decentralized confirmatory testing can be performed.

Our results are limited by the assumption that mean annual suspected cholera incidence estimates from 2010 to 2016 (ref. ^1^) represent a static risk of cholera during our projection period. These estimates depend on reporting and care-seeking for cholera symptoms and may not accurately reflect current and future burden. Consequently, it is more appropriate to interpret the relative magnitude of scenarios rather than the absolute public health impact or costs or the results of any single country. Second, our model projects average burden trends over time, without consideration of high annual variability in cholera transmission (for example, due to outbreaks or humanitarian emergencies). Finally, to narrow the scope of our investigation, we applied highly simplified approaches to vaccine targeting, based only on incidence rates and without vaccine supply constraints, as well as to vaccine efficacy, where there were no age-specific differences and all vaccinated individuals received two doses.

Robust disease surveillance has long been cited as essential for efficient vaccine targeting^22,23^, but other advantages would follow as well. Facilitated by the short turnaround time of RDTs, decentralized testing in cholera-outbreak-prone settings could enhance the rollout of control activities such as reactive vaccination and case-area targeted interventions, which would have otherwise relied on suspected case data for prioritization^24^. A 2023 update to GTFCC guidance now recommends countries to prioritize areas for interventions including preventive vaccination through a multidimensional index that includes cholera test positivity from RDTs, culture or PCR, in addition to suspected case incidence, persistence and mortality^25^. Both financial and political investments are needed by ministries of health and the broader global health community to translate diagnostic development into effective surveillance and vaccine distribution for cholera control.

## Methods

Here we describe the model input data and parameters, the components of targeting scenarios, the simulation framework, and calculation of public health and cost-effectiveness metrics.

### Model inputs

#### Suspected cholera incidence rates

Previously published gridded estimates of mean suspected cholera incidence rate from 2010 to 2016 in 35 countries were downscaled from 20 km × 20 km to 5 km × 5 km by assuming that 5 km × 5 km cells had the same rate as the overlapping 20 km × 20 km cell^1^. We assumed that these rates would remain constant from 2022 through 2035 in the absence of modeled vaccination, but the number of cases could change as the population size changed each year. These gridded estimates were then aggregated to an administrative unit scale as the population-weighted mean of each of its grid cells.

We assumed that a variable fraction of suspected cholera case incidence was due to true cholera infections. To simulate the incidence rate of ‘true cholera’ we multiplied the suspected incidence rate in each administrative unit by a positivity proportion (‘*V. cholerae* positivity’). *V. cholerae* positivity was drawn randomly for each administrative unit and simulation, but was assumed constant across years and modeling scenarios. We assumed that *V. cholerae* positivity followed a beta distribution (*α* = 5.604821 and *β* = 5.125101) that was fit to 1,000 posterior predictive samples of the pooled adjusted *V. cholerae* positivity from a recent meta-analysis^12^.

We defined administrative areas according to the Global Administrative area database (GADM) using the R package GADMtools (version 3.9.1)^26^. Hereafter, first-level administrative units are called ‘provinces’ and second-level administrative units are called ‘districts’.

#### Vaccine properties

As in a previous modeling study^6^, we assumed that complete vaccination had a direct protective effect of 66% in the first year, which waned to 0% after 5 years^27^. Indirect vaccine effects were modeled as a relative (multiplicative) reduction in incidence rate for unvaccinated individuals according to the corresponding grid cell’s vaccination coverage. We assumed 68% of individuals 1 year old and above in the targeted administrative unit received two doses of vaccine in the vaccination year and otherwise none, similar to a previous study^6^.

#### Population data

Annual country population estimates and projections were from UN World Population Prospects^28^. The spatial distribution of population within a country followed the relative population proportions of the unconstrained 2020 1 km × 1 km WorldPop population raster after it was aggregated to the 5 km × 5 km resolution^29,30^.

### Modeling scenarios

We explored the potential impact and efficiency of targeted cholera vaccine use in scenarios that varied by three primary variables: bacteriological confirmation capacity (three settings), incidence rate thresholds (three levels, above which administrative units would be targeted for vaccination) and administrative scale of the vaccination campaign targeting (two scales). In total, we simulated 18 vaccination scenarios to represent all combinations (3 × 3 × 2) and one ‘no vaccination’ scenario (Fig. 1).

#### Bacteriological confirmation capacity

Bacteriological confirmation capacity represents a country’s capacity for systematic confirmatory testing. This setting determines how well the incidence rates ‘observed’ by the surveillance system align with true incidence of *V. cholerae*; the observed incidence rate determined where vaccines were allocated.

We considered three bacteriological confirmation capacity scenarios. The low-capacity setting, ‘clinical definition’, assumed that only suspected incidence rates were observed as no systematic confirmatory testing was performed (representing the current practice in much of the world). To calculate testing costs, the ‘decentralized testing’ setting assumed that suspected cholera samples were tested systematically with RDTs and RDT-positive samples were tested systematically with culture in district-level laboratories, following Global Task Force for Cholera Control (GTFCC) public health surveillance guidance in confirmed outbreak settings (see ‘How testing works’ section and Extended Data Table 3)^8^. In this setting, the observed and true *V. cholerae* incidence rates were assumed to be the same. The primary results compared scenarios with ‘clinical definition’ and ‘decentralized testing’ for district-level campaign targeting.

Both decentralized and centralized testing settings use *V. cholerae* positivity estimates that have already been adjusted for the sensitivity and specificity of different test types (see more in ‘How testing works’ section)^12^. The ‘centralized testing’ setting assumes that suspected cholera samples are tested systematically with culture in a national reference laboratory when calculating testing costs^17^ These tests experience an additional 20% reduction in test sensitivity compared to the decentralized testing setting, an attenuation that is motivated by testing delays and damage to samples that may occur as samples are delivered to a reference laboratory. In this setting, the observed incidence rate was roughly 20% lower than the true *V. cholerae* incidence rate at the national level, with variation derived from district-level *V. cholerae* positivity (see ‘Suspected cholera incidence rates’ section).

#### Targeting thresholds

Administrative units were vaccinated if their observed incidence rate exceeded the threshold of 10 cases per 10,000 population, 2 cases per 10,000 population or 1 case per 10,000 population, according to the scenario.

#### Administrative scale of the vaccination campaign

Vaccination campaign targets were identified at the district or province level of the country. In scenarios with province-level targeting, the province *V. cholerae* positivity was calculated as the suspected-case-weighted mean of the associated district-level *V. cholerae* positivities.

### How testing works

We considered three bacteriological confirmation capacity settings in our scenarios, each representing a different way to determine the observed incidence rate in a given administrative unit. The observed incidence rate was calculated as the product of a suspected cholera incidence rate and a *V. cholerae* positivity; in the case of the ‘clinical definition’ setting, the suspected cholera and observed incidence rates were the same as no systematic testing was applied. In the ‘decentralized testing’ setting, we drew a random value from a distribution of *V. cholerae* positivity estimates for each simulation and second-level administrative unit (district). To make the ‘centralized testing’ setting comparable to the corresponding ‘decentralized testing’ setting, we calculated the mean positivity of each simulation across all districts in the decentralized testing scenario and applied it to all districts in the centralized testing scenario. In addition, we applied a 20% reduction to this mean positivity to reflect sensitivity loss from testing delays and damage to samples that may occur as samples are delivered to a centralized reference laboratory. For all testing scenarios, positivity remained the same for all modeled years, but differed across administrative units (in decentralized testing) and simulations.

The *V. cholerae* positivity distribution was derived from Wiens et al.^12^. They first conducted a systematic review of qualified studies from 2000 to 2023 that tested suspected cholera cases using either culture, PCR or RDTs. Based on data from four studies that used all three diagnostic tests, they constructed a hierarchical conditional dependence model under a Bayesian framework to estimate each diagnostic test’s sensitivity and specificity. Next, these estimated diagnostic-test-specific sensitivities and specificities were used to adjust for the possibility of false negatives and false positives to arrive at an overall adjusted positivity estimate. This positivity estimate was pooled across all 119 included studies and fit in a generalized linear model with study-level random effects. They estimated that on average 52% (95% credible interval 24–80%) of suspected cholera cases were true *V. cholerae* infections. We fit 1,000 posterior samples of this adjusted positivity estimate to a beta distribution (*α* = 5.604821 and *β* = 5.125101) and used the parametric distribution to draw random *V. cholerae* positivity estimates.

While positivity could vary as a function of observed incidence, Wiens et al. found that *V. cholerae* positivity was not associated with the estimated suspected cholera 2010–2016 mean annual incidence rate^1^ in the administrative unit containing each study’s research site. Consequently, we assumed in our models that *V. cholerae* positivity was incidence-independent.

### How targeting works

For each year in the projection period, the model alternates between a vaccine targeting step and an epidemiologic modeling step (Extended Data Table 1). OCV targeting is performed dynamically in each projection year on the basis of the observed mean annual incidence rate in a given administrative unit over the past 5 years; this dynamic targeting differs from the originally published model^6^.

In brief, the model begins with a single simulation of the gridded 2010–2016 mean annual suspected cholera incidence rate^1^. Processes that affect population susceptibility to disease (that is, direct and indirect vaccine effects, vaccination campaign coverage and natural population turnover) were then applied. All administrative units with an observed 5-year mean annual incidence rate greater than the targeting threshold (that is, 10 cases, 20 cases or 1 case per 10,000 population) were vaccinated, such that 68% percent of the population aged 1 year and older received two vaccine doses.

After the vaccine targeting and epidemiologic modeling steps, a new 5-year observed mean annual incidence rate is calculated to inform the vaccine targeting step in the next projection year.

### Epidemiologic model assumptions

Assumptions related to direct and indirect vaccine effects, vaccination campaign coverage and population turnover were the same as a previously published paper^7^.

For direct vaccine effects, we estimated annual vaccine efficacy in years 0 through 5 after vaccination by taking the mean point estimates from a fitted log-linear model fit to pooled two-dose vaccine efficacy estimates identified in a systematic review and meta-analysis of OCVs^6^. The initial vaccine efficacy was 66% in the year of vaccination, then declined to 0% in year 6.

For indirect vaccine effects, we fit a logistic function to data gathered from India and Bangladesh on the association between the relative reduction in the incidence among unvaccinated individuals in the OCV-coverage neighborhood^6^. Fitted values from the logistic function were then applied as a ‘percent reduction in incidence due to vaccination-induced immunity’ at the grid-cell level of the model. With this assumption, unvaccinated individuals received 80% and almost 100% mitigation in cholera incidence if their neighborhood (simulated as the 5 km by 5 km grid cell in the model) had a 50% or 70% vaccination coverage, respectively.

Vaccination campaign coverage was assumed to be 68% on the basis of a review of published data from seven vaccination campaign coverage surveys. Additional details and the extracted survey data may be found in the previously published paper^6^.

Population turnover represents the loss of vaccinated individuals in the population due to births and deaths since the time of the last vaccination campaign. As in the original paper, country-specific life expectancy in the model year and number of years since the last vaccination campaign were used to calculate the proportion of the model year population that retained their vaccinated status^6^.

### Model equations

We modeled each country and scenario independently. For each model year in the simulation, there were alternating vaccine targeting and epidemiologic modeling steps.

#### Targeting vaccines on the basis of observed incidence rates

Vaccines were targeted on the basis of the observed mean annual incidence rate in administrative level 2 units (districts) in the previous 5-year period. The observed mean annual incidence rate (${\lambda }_{i,p,s}^{{{\mathrm{obs}}}}$) depended on the bacteriological confirmation capacity setting (Fig. 1, bacteriological confirmation capacity).

In the clinical definition setting:

$${\lambda }_{i,p,s}^{{{\mathrm{obs}}}}={\lambda }_{i,p,s}^{{{\mathrm{sus}}}},$$

where ${\lambda }_{i,p,s}^{{{\mathrm{sus}}}}$ is the mean annual incidence rate of suspected cholera in district *i*, in the previous 5-year period *p*, and simulation *s*. The observed and suspected incidence rates were equivalent because no systematic testing was performed; therefore, only suspected incidence rates were available for use in vaccine targeting. The suspected cholera incidence rate for a district was the population-weighted mean of the associated 5 km by 5 km gridded estimates of mean annual suspected cholera incidence:

$${\lambda }_{i,p,s}^{{{\mathrm{sus}}}}=\sum _{j\in i}{\lambda }_{j,p,s}^{{{\mathrm{sus}}}}\times \frac{{{{\mathrm{pop}}}}_{j}}{{{{\mathrm{pop}}}}_{i}},$$

where $j\in i$ represents the set of *j* 5 km by 5 km grid cells that overlap with the *i* district. The gridded estimates were derived from previously published 20 km by 20 km gridded estimates of mean annual suspected cholera incidence in 2010–2016 across Africa^1^; all 5 km by 5 km grid cells in the same 20 km by 20 km grid cell had the same incidence rate $\lambda$.

In the decentralized testing setting:

$${\lambda }_{i,p,s}^{{{\mathrm{obs}}}}={\lambda }_{i,p,s}^{{{\mathrm{true}}}},$$

where ${\lambda }_{i,p,s}^{{{\mathrm{true}}}}$ is the unobserved, underlying true cholera mean annual incidence rate. We assumed that a fraction of suspected cholera cases were true cholera cases, such that

$${\lambda }_{i,p,s}^{{{\mathrm{true}}}}={\lambda }_{i,p,s}^{{{\mathrm{sus}}}}\times {\alpha }_{i,s}^{{{\mathrm{true}}}},$$

where ${\alpha }_{i,s}^{{{\mathrm{true}}}}$ is the underlying true *V. cholerae* positivity that remains constant across all years for a given district and simulation (Fig. 1, model input). We assumed that decentralized testing would return perfect estimation of the true positivity ${\alpha }_{i,s}^{{{\mathrm{true}}}}$, and thus underlying incidence rates were perfectly observed.

In the centralized testing setting:

$${\lambda }_{i,p,s}^{{{\mathrm{obs}}}}={\lambda }_{i,p,s}^{{{\mathrm{sus}}}}\times {\alpha }_{N,s}\times (1-\psi ),$$

where $\psi$ is the loss of sensitivity during shipment of samples to the national reference laboratory, and ${\alpha }_{N,s}$ is the *V. cholerae* positivity for country *N*. This is calculated as the suspected-case-weighted mean of all true *V. cholerae* positivity values at the district level:

$${\alpha }_{N,s}=\sum _{i\in N}{\alpha }_{i,s}^{{{\mathrm{true}}}}\times \frac{{\lambda }_{i,s}^{{{\mathrm{sus}}},y1}\times {{{\mathrm{pop}}}}_{i}}{{\lambda }_{N,s}^{{{\mathrm{sus}}},y1}\times {{{\mathrm{pop}}}}_{N}},$$

where $i\in N$ represents all districts in country *N*. A country’s *V. cholerae* positivity (${\alpha }_{N,s}$) was constant for all model years; the suspected-case-weighted-mean for only the first model year (*y*1) was used in this calculation.

All districts *i* with an observed mean annual incidence rate (${\lambda }_{i,p,s}^{{{\mathrm{obs}}}}$) that exceeded a scenario’s incidence rate targeting threshold and had not been targeted by a campaign within the last three years were targeted with a vaccination campaign in a given scenario (Fig. 1, identifying OCV targets for each simulation year).

#### Epidemiologic modeling to update true incidence rates

Epidemiologic modeling occurs at the grid cell level. For all districts selected for vaccination in a given year, we calculated the proportion of the population vaccinated in each 5 km by 5 km grid cell:

$${v}_{j,y,s}=\theta \times {{{\mathrm{pop}}}}_{j,y}\times \frac{{{{\mathrm{pop}}}}_{N,y}^{{{\mathrm{age}}}\ge 1}}{{{{\mathrm{pop}}}}_{N,y}},$$

where $\theta$ is the vaccination campaign coverage proportion, *y* is the model projection year, and ${{{\mathrm{pop}}}}_{N,y}^{{{\mathrm{age}}}\ge 1}$ is the number of people in the country projected to be 1 year old and above according to UN World Population Prospects^27^. Only individuals aged 1 year and above are eligible for oral cholera vaccination. For all admin 2 units not selected for vaccination, ${v}_{j,y,s}=0$.

The proportion of the population susceptible to infection (that is, those not protected by vaccine) (${\sigma }_{j,y,s}$) is a function of vaccination campaigns in the current year and previous 5 years:

$${\sigma }_{j,y,s}=\mathop{\prod }\limits_{m=y-5}^{y}(1-(v_{j,m,s}\times {\delta }_{y-m}\times {\tau }_{j,m\to y})),$$

where ${v}_{j,m,s}$ is the proportion of the *j* grid cell population vaccinated in year *m*, ${\delta }_{y-m}$ is the proportion protected from direct vaccine effects in *y*–*m* years since vaccination, and ${\tau }_{j,m\to y}$ is the proportion of the population from year *m* that remains in the population in year *y* after accounting for population turnover. The population turnover multiplier (${\tau }_{j,m\to y}$) is calculated as

$${\tau }_{j,m\to y}={{{\mathrm{pop}}}}_{j,m}\times (1-((\,y-m)\times {\mu }_{N,y}))/{{{\mathrm{pop}}}}_{j,y},$$

where ${\mu }_{N,y}$ is the inverse of the life expectancy in country *N* and year *y* according to UN World Population Prospects^28^.

The gridded true expected cholera cases in the next model year (${\varepsilon }_{j,y+1,s}^{{{\mathrm{true}}}}$) is updated in the epidemiologic model as

$${\varepsilon }_{j,y+1,s}^{{{\mathrm{true}}}}={{{\mathrm{pop}}}}_{j,y}\times {\lambda }_{i,s}^{{{\mathrm{true}}},y1}\times {\sigma }_{j,y,s}\times {\iota }_{j,y,s},$$

where ${\lambda }_{i,s}^{{{\mathrm{true}}},y1}$ represents the true underlying incidence rate in the first model year and remains constant for all simulation years; this assumes that cholera risk remains static over time. The ${\iota }_{j,y,s}$ represents the reduction in population susceptibility due to indirect vaccine effects, which are a function of grid cell *j* vaccine-derived protection in year *y* (see more in ‘Epidemiologic model assumptions’ section). Consequently, the true incidence rate at the district level (${\lambda }_{i,y+1,s}^{{{\mathrm{true}}}}$) can be calculated as

$${\lambda }_{i,y+1,s}^{{{\mathrm{true}}}}=\sum _{j\in i}{\varepsilon }_{j,y+1,s}^{{{\mathrm{true}}}}\times \frac{1}{{{{\mathrm{pop}}}}_{j,y+1}}.$$

The suspected cholera incidence rate for the next model year (${\lambda }_{i,y+1,s}^{{{\mathrm{sus}}}}$) is then back-calculated from the true incidence rate such that

$${\lambda }_{i,y+1,s}^{{{\mathrm{sus}}}}={\lambda }_{i,y+1,s}^{{{\mathrm{true}}}}/{\alpha }_{i,s}^{{{\mathrm{true}}}}.$$

The vaccine targeting step then begins for the next modeled year.

#### Targeting vaccine to administrative level 1 units (provinces)

While the primary manuscript scenarios targeted vaccine to districts, we also ran analogous model scenarios when vaccine was targeted to administrative level 1 units (provinces).

In the clinical definition setting, model equations for province-level targeting mirrored those for district-level targeting:

$${\lambda }_{k,p,s}^{{{\mathrm{obs}}}}={\lambda }_{k,p,s}^{{{\mathrm{sus}}}}$$

and

$${\lambda }_{k,p,s}^{{{\mathrm{sus}}}}=\sum _{j\in k}{\lambda }_{j,p,s}^{{{\mathrm{sus}}}}\times \frac{{{{\mathrm{pop}}}}_{j}}{{{{\mathrm{pop}}}}_{k}},$$

where $j\in k$ represents the set of *j* 5 km by 5 km grid cells that overlap with the *k* province.

In the decentralized testing setting, the true and observed mean annual incidence rates in period *p* for vaccine targeting mirrored those for district-level targeting:

$${\lambda }_{k,p,s}^{{{\mathrm{obs}}}}={\lambda }_{k,p,s}^{{{\mathrm{true}}}}$$

and

$${\lambda }_{k,p,s}^{{{\mathrm{true}}}}={\lambda }_{k,p,s}^{{{\mathrm{sus}}}}\times {\alpha }_{k,s}^{{{\mathrm{true}}}},$$

and *V. cholerae* positivity at the province level (${\alpha }_{k,s}^{{{\mathrm{true}}}}$) was calculated as

$${\alpha }_{k,s}^{{{\mathrm{true}}}}=\sum _{i\in k}{\alpha }_{i,s}^{{{\mathrm{true}}}}\times \frac{{\lambda }_{i,s}^{{{\mathrm{sus}}},y1}\times {{{\mathrm{pop}}}}_{i}}{{\lambda }_{k,s}^{{{\mathrm{sus}}},y1}\times {{{\mathrm{pop}}}}_{k}}$$

where $i\in k$ represents all districts *i* in province *k*. Province-level *V. cholerae* positivity remained constant across all years and relied only on district suspected-case weights from the first model year ($y1$).

In the centralized testing setting,

$${\lambda }_{k,p,s}^{{{\mathrm{obs}}}}={\lambda }_{k,p,s}^{{{\mathrm{sus}}}}\times {\alpha }_{N,s}\times (1-\psi ),$$

and the country-level *V. cholerae* positivity (${\alpha }_{N,s}$) was calculated as

$${\alpha }_{N,s}=\sum _{i\in N}{\alpha }_{i,s}^{{{\mathrm{true}}}}\times \frac{{\lambda }_{i,s}^{{{\mathrm{sus}}},y1}\times {{{\mathrm{pop}}}}_{i}}{{\lambda }_{N,s}^{{{\mathrm{sus}}},y1}\times {{{\mathrm{pop}}}}_{N}},$$

the same as that for the district-targeting scenarios, which makes the district and province targeting scenarios more comparable.

### Model simulation

We performed simulations independently by country for a duration that enabled three possible rounds of vaccination campaigns (2022–2030) and five additional years for waning vaccine effects (2031–2035). The timeline was chosen to mirror the 2030 cholera control targets set by the 71st World Health Assembly^31^. We modeled all countries in Africa where spatial clinical cholera incidence estimates were available, which included all cholera-affected African countries identified in the GTFCC Global Roadmap to End Cholera^31^. Thirty-five countries were modeled: Angola, Burundi, Benin, Burkina Faso, Central African Republic, Côte d’Ivoire, Cameroon, the Democratic Republic of the Congo, Republic of the Congo, Ethiopia, Ghana, Guinea, Guinea-Bissau, Kenya, Liberia, Madagascar, Mali, Mozambique, Mauritania, Malawi, Namibia, Niger, Nigeria, Rwanda, Senegal, Sierra Leone, Somalia, South Sudan, Chad, Togo, Tanzania, Uganda, South Africa, Zambia and Zimbabwe.

For scenarios with vaccination, each simulation year proceeds with vaccine targeting and epidemiologic modeling steps during the 2022–2030 vaccination period, and with only epidemiologic steps from 2031 to 2035 (Extended Data Table 1). All vaccinated individuals were assumed to be fully vaccinated with two doses. Simulations of the no-vaccination scenario included only the epidemiologic step.

### Model outputs

#### Public health impact

We calculated true cholera cases averted as the difference between true cholera cases in matched vaccination and no-vaccination simulations. OCV campaign efficiency was defined as the number of true cholera cases averted per 1,000 FVPs. Relative efficiency was the ratio of OCV campaign efficiencies in two vaccination scenarios. Percent of FVPs living in high-incidence-rate administrative units is calculated by dividing FVP living in units with a true incidence rate that exceeded the scenario’s targeting threshold by the total FVP in the scenario.

#### Number of tests performed

We followed the GTFCC public health surveillance guidance to determine how many and what kinds of tests were performed under the decentralized and centralized testing setting^8^. The decentralized testing setting assumed that the first three suspected cases per health facility per day were tested with RDTs and the first three RDT-positive cases per week per surveillance unit were tested with culture (Extended Data Table 3). The centralized testing setting assumed that the first three suspected cases per health facility per week were tested with culture (Extended Data Table 3). The percentage of suspected cases tested in the two settings was based on daily clinical surveillance data in four health facilities in endemic cholera transmission regions in the Democratic Republic of the Congo and Bangladesh. We assumed that no tests were performed in the clinical definition setting.

#### Testing and vaccination costs

Based on expert consultation and a brief review of reagent costs from major brands, we assumed that the cost of performing RDT and culture was US$1.9 and US$13 per test, respectively (Extended Data Tables 4 and 5)^10,32^. We assumed that the cost of procuring, shipping, and delivering OCV was US$2.36 per dose (Extended Data Table 4)^11–13,33–35^. Total costs for a scenario refer to the sum of testing and OCV costs for all tests and doses administered.

Cost-effectiveness was calculated as total cost per averted case. To understand tradeoffs in testing and vaccination with the introduction of systematic decentralized testing, we subtracted OCV cost per averted case and test cost per averted case between the clinical definition and decentralized testing scenarios. This subtraction yielded two metrics, OCV cost reduction per averted case and test cost spent per averted case, respectively. OCV cost reduction per test dollar spent is the ratio of OCV cost reduction per averted case and test cost per averted case.

### Statistical analysis

For each scenario, we joined the 200 country-level simulations to create 200 continent-wide simulations and calculated median and PIs across the continent-wide scenario results. PIs around these metrics represent stochasticity that was introduced through selection of 200 random posterior draws of mean annual suspected cholera incidence rate maps and random draws from the *V. cholerae* positivity distribution (by district and suspected cholera incidence draw)^12^. A random seed was fixed to enable direct comparison of simulations across modeling scenarios. For a given metric, scenarios with non-overlapping 95% PIs were considered to be statistically significantly different.

### Ethics and inclusion statement

Data for this study includes previously published estimates of cholera burden in Africa. The original study included co-authorship for and feedback from African country representatives that contributed confidential data to the modeling effort.

For this current work, we do not encourage country-specific interpretation of the results due to the strategic nature of the research question; complete scenario-based comparisons are more appropriate for interpretation. Rather, the research is locally relevant to the studied countries in that it compares different surveillance testing strategies and their effects on targeting cholera vaccine. These results provide local decision-makers with data on the relative benefits of enhanced cholera surveillance, thus motivating possible changes in national surveillance guidelines.

Three authors (E.B.M., G.B. and P.W.O.) are based in cholera-affected low- and middle-income countries (LMICs), and provided feedback on the interpretation and application of this work based on their expertise in cholera surveillance in LMIC contexts. We fully endorse the Nature Portfolio journals’ guidance on LMIC authorship and inclusion, and are strongly committed to the inclusion of more researchers and decision-makers from LMICs in future related work.

### Reporting summary

Further information on research design is available in the Nature Portfolio Reporting Summary linked to this article.

## Online content

Any methods, additional references, Nature Portfolio reporting summaries, source data, extended data, supplementary information, acknowledgements, peer review information; details of author contributions and competing interests; and statements of data and code availability are available at 10.1038/s41591-024-02852-8.

### Supplementary information

Reporting Summary

## Figures and Tables

**Extended Data Table 1:**
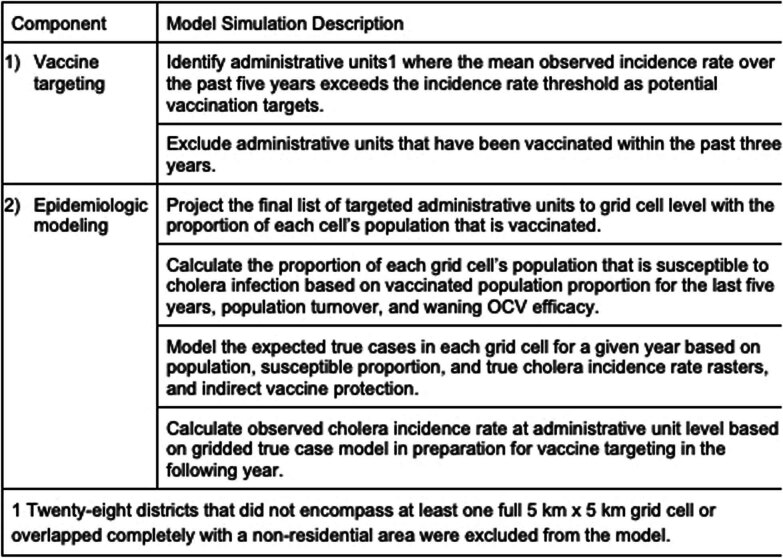
Description of model simulation steps in vaccination scenarios

The two model components, vaccine targeting and epidemiologic modeling, are implemented in sequential order for each year of vaccination campaigns from 2022-2030. Only the epidemiologic modeling component is implemented in simulation years 2031-2035 to simulate the impact of waning vaccine protection and population turnover after the end of campaigns. A summary of steps within each component is provided.

**Extended Data Table 2:**
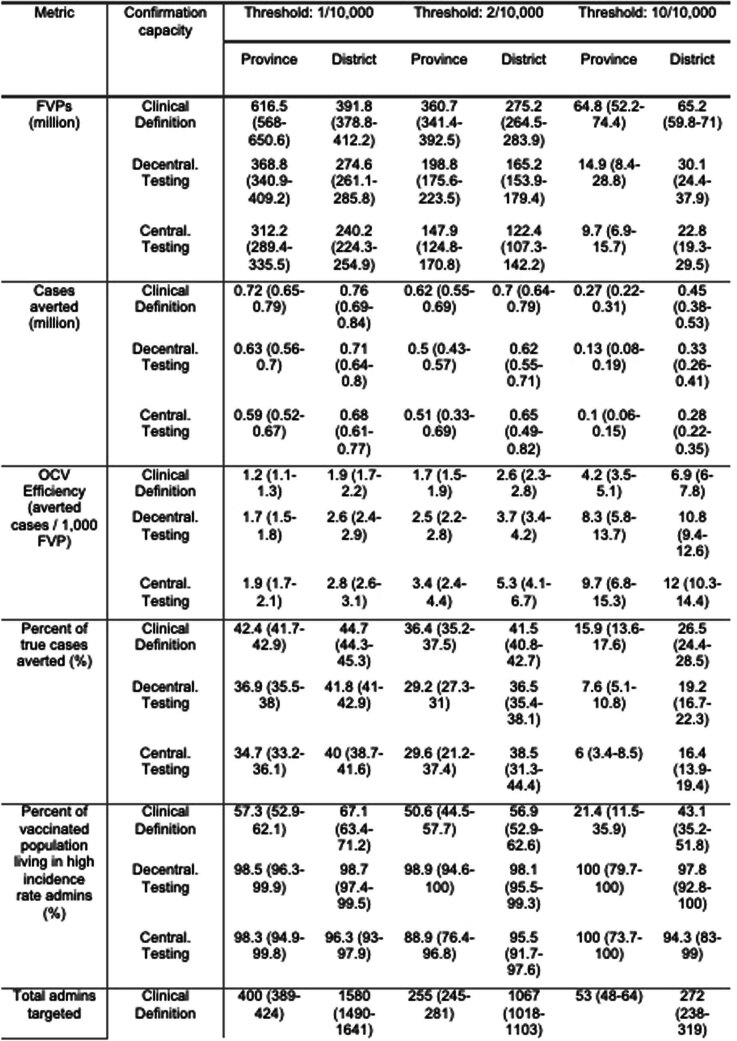
Model output metrics for all scenarios, 2022-2035

For all 18 vaccination modeling scenarios, which vary by targeting threshold, campaign targeting scale, and bacteriological confirmation capacity, we report the median estimates and 95% prediction intervals in parentheses for number of fully vaccinated persons (FVP) in millions, true cholera cases averted in millions (averted cases), proportion of true cases not averted in percentage, and true cases averted per 1,000 FVP (OCV efficiency), proportion of FVP living in high incidence rate areas in percentage, total (with repetition) and unique (without repetition) number of administrative units and countries targeted by the OCV campaigns. Only district targeting scale results are reported in the main text.

**Extended Data Table 3:**
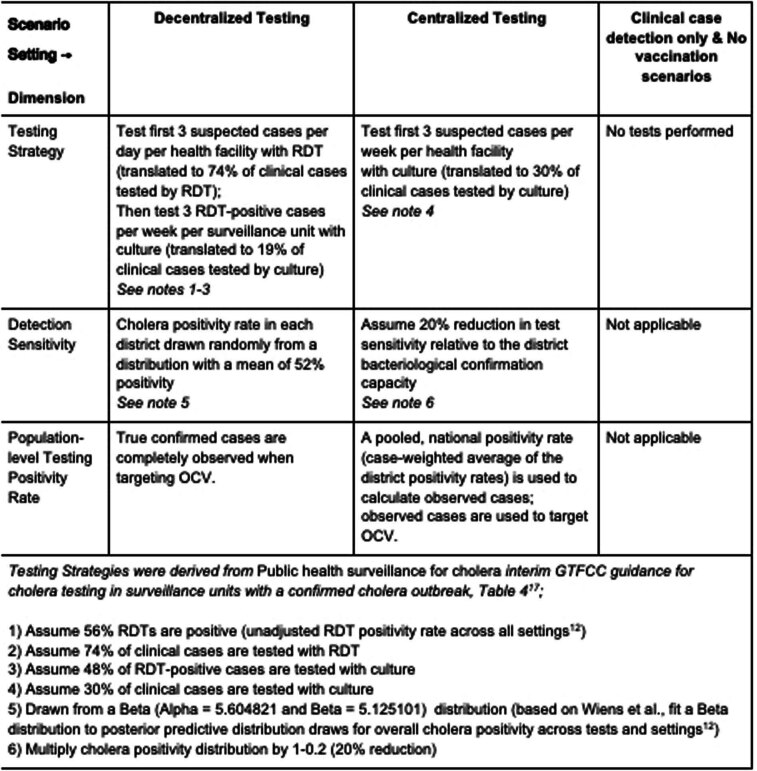
Differences in bacteriological confirmation capacity settings

This table describes how the three settings differ in terms of systematic testing strategy and number of tests performed, detection sensitivity, and population-level positivity rate.

**Extended Data Table 4:**
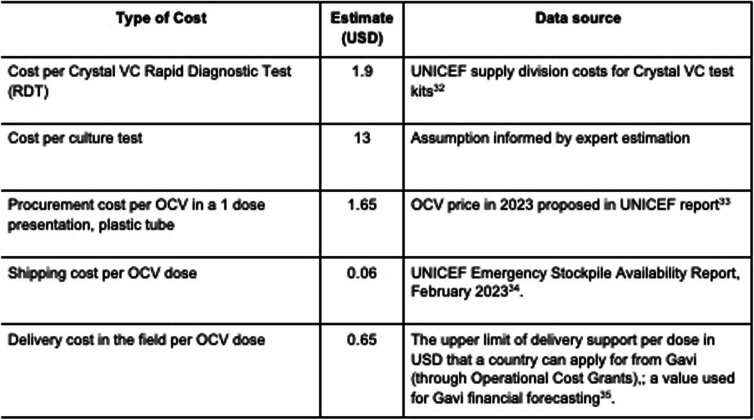
Testing and OCV campaign cost assumptions

**Extended Data Table 5:**
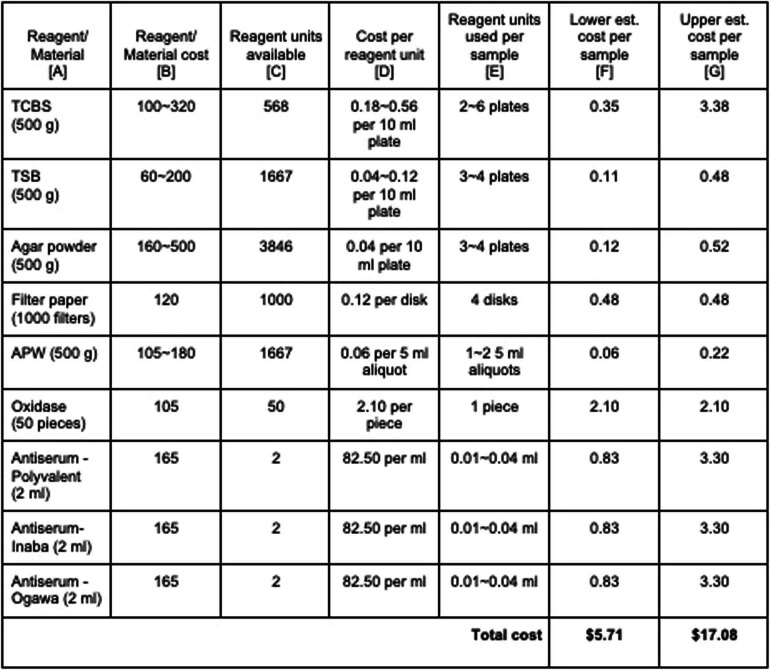
Estimated cost of V. cholerae culture in US dollars (USD)

This table includes rounded, estimated costs of reagents and materials required to perform culture of *V. cholerae* from suspected case stool samples stored in agar stabs, as solicited by expert information. Our model assumption of $13 USD per sample falls within the range presented in the table. The costs listed in [B] are the estimated total costs for a box or bottle of reagent or material. The term ‘reagent unit’ [C-E] refers to the number of preparations that can be made from the amount of reagent or material listed in [A]. Cost per reagent unit [D] is calculated as the Reagent/ Material cost [B] divided by Reagent units available [C]. In some cases, multiple preparations may be required to test a single sample [E]. Lower and upper estimated costs per sample [F-G] are calculated as the product of Cost per reagent unit [C] and Reagent units used per sample [E]. These estimates do not take into account the costs of plastic consumables or running a laboratory.

## Data Availability

Summary model outputs related to vaccine targeting, public health impact and cost-effectiveness for each scenario are available on figshare at 10.6084/m9.figshare.25037177. Subnational shapefiles are available from the GADM, which we accessed from the R package GADMtools (version 3.9.1).

## Code availability

All modeling and analyses were performed in R version 4.0.3 14 (ref. ^36^). The model code, inputs, and explanatory README setup file can be accessed in the Github repository at https://github.com/HopkinsIDD/gavi_vimc_cholera.
