# Author Correction: Enhanced cholera surveillance to improve vaccination campaign efficiency

**DOI:** 10.1038/s41591-024-03176-3

**Published:** 2024-07-09

**Authors:** Hanmeng Xu, Kaiyue Zou, Juan Dent, Kirsten E. Wiens, Espoir Bwenge Malembaka, Godfrey Bwire, Placide Welo Okitayemba, Lee M. Hampton, Andrew S. Azman, Elizabeth C. Lee

**Affiliations:** 1 Department of Epidemiology, Johns Hopkins Bloomberg School of Public Health, Baltimore, MD USA; 2 Department of Epidemiology and Biostatistics, College of Public Health, Temple University, Philadelphia, PA USA; 3 Center for Tropical Diseases and Global Health, Université Catholique de Bukavu, Bukavu, Democratic Republic of the Congo; 4 Division of Public Health Emergency Preparedness and Response, Ministry of Health, Kampala, Uganda; 5 Makerere University School of Public Health, Kampala, Uganda; 6 Programme National d’Elimination de Choléra et lutte contre les autres Maladies Diarrhéiques, Kinshasa, Democratic Republic of the Congo; 7 Gavi, the Vaccine Alliance, Geneva, Switzerland; 8 Geneva Centre for Emerging Viral Diseases, Geneva University Hospitals, Geneva, Switzerland; 9 Division of Tropical and Humanitarian Medicine, Geneva University Hospitals, Geneva, Switzerland

**Keywords:** Bacterial infection, Computational models

Correction to: *Nature Medicine* 10.1038/s41591-024-02852-8, published online 5 March 2024.

In the version of the article initially published, National Institutes of Health grant K22AI168389 was missing from the Acknowledgements and has now been added to the HTML and PDF versions of the article.

