# Supplementary material for: Enhanced cholera surveillance to improve vaccination campaign efficiency
Source: Nat Med. 2024 Mar 5;30(4):1104–10. doi: 10.1038/s41591-024-02852-8 (PMC11031394; doi:10.1038/s41591-024-02852-8)
Supplement: Supplementary file 1 — Reporting Summary [file 41591_2024_2852_MOESM1_ESM.pdf]

nature portfolio

Corresponding author(s): Elizabeth Lee

Last updated by author(s): 1/18/2024

Reporting Summary

Nature Portfolio wishes to improve the reproducibility of the work that we publish. This form provides structure for consistency and transparency in reporting. For further information on Nature Portfolio policies, see our Editorial Policies and the Editorial Policy Checklist.

Statistics

For all statistical analyses, confirm that the following items are present in the figure legend, table legend, main text, or Methods section.

n/a	Confirmed	
☐	☒	The exact sample size (n) for each experimental group/condition, given as a discrete number and unit of measurement
☒	☐	A statement on whether measurements were taken from distinct samples or whether the same sample was measured repeatedly
☒	☐	The statistical test(s) used AND whether they are one- or two-sided<br>*Only common tests should be described solely by name; describe more complex techniques in the Methods section.*
☐	☒	A description of all covariates tested
☐	☒	A description of any assumptions or corrections, such as tests of normality and adjustment for multiple comparisons
☐	☒	A full description of the statistical parameters including central tendency (e.g. means) or other basic estimates (e.g. regression coefficient) AND variation (e.g. standard deviation) or associated estimates of uncertainty (e.g. confidence intervals)
☒	☐	For null hypothesis testing, the test statistic (e.g. F, t, r) with confidence intervals, effect sizes, degrees of freedom and P value noted<br>*Give P values as exact values whenever suitable.*
☒	☐	For Bayesian analysis, information on the choice of priors and Markov chain Monte Carlo settings
☒	☐	For hierarchical and complex designs, identification of the appropriate level for tests and full reporting of outcomes
☒	☐	Estimates of effect sizes (e.g. Cohen's d, Pearson's r), indicating how they were calculated

Our web collection on statistics for biologists contains articles on many of the points above.

Software and code

Policy information about availability of computer code

Data collection	Model inputs and assumptions are available at https://github.com/HopkinsIDD/gavi_vimc_cholera
Data analysis	Modeling was conducted in R version 4.0.3 and all code is publicly available at https://github.com/HopkinsIDD/gavi_vimc_cholera

For manuscripts utilizing custom algorithms or software that are central to the research but not yet described in published literature, software must be made available to editors and reviewers. We strongly encourage code deposition in a community repository (e.g. GitHub). See the Nature Portfolio guidelines for submitting code & software for further information.

Data

Policy information about availability of data

All manuscripts must include a data availability statement. This statement should provide the following information, where applicable:

- Accession codes, unique identifiers, or web links for publicly available datasets
- A description of any restrictions on data availability
- For clinical datasets or third party data, please ensure that the statement adheres to our policy

Summary model outputs related to vaccine targeting, public health impact, and cost-effectiveness for each scenario are available on figshare at https://doi.org/10.6084/m9.figshare.25037177. Sub-national shapefiles are available from the Global Administrative area database (GADM), which we accessed from the R package GADMtools (version 3.9.1).

Human research participants

Policy information about studies involving human research participants and Sex and Gender in Research.

Reporting on sex and gender	Not applicable
Population characteristics	Not applicable
Recruitment	Not applicable
Ethics oversight	Not applicable

Note that full information on the approval of the study protocol must also be provided in the manuscript.

Field-specific reporting

Please select the one below that is the best fit for your research. If you are not sure, read the appropriate sections before making your selection.

☒ Life sciences ☐ Behavioural & social sciences ☐ Ecological, evolutionary & environmental sciences

For a reference copy of the document with all sections, see nature.com/documents/nr-reporting-summary-flat.pdf

Life sciences study design

All studies must disclose on these points even when the disclosure is negative.

Sample size	No sample size calculation was performed. There are no "samples" in this modeling study. We chose to perform 200 stochastic simulations for each modeled scenario to capture variability in the distribution of the estimate of the mean annual incidence rate and the distribution of the cholera positivity rate. Since there are only two sources of stochastic variability in our model, we thought 200 samples was sufficiently large to explore the parameter space while remaining computationally tractable.
Data exclusions	No data were excluded from the analysis.
Replication	200 stochastic simulations were performed for each modeling scenario. Our reported results represent summary statistics across 200 stochastic simulations. Here, we are not looking for results to be replicated across different simulations, but in reporting the summary statistics across many simulations we are trying to understand trends that hold true across a distribution of model parameters.
Randomization	This is not relevant to our modeling study. The study uses model assumptions and model simulation to test hypotheses. We did not aim to test the effectiveness of an intervention in different study populations using real-world data.
Blinding	This is not relevant to our modeling study. There were no patients or intervention arms in our modeling study so blinding is not relevant.

Reporting for specific materials, systems and methods

We require information from authors about some types of materials, experimental systems and methods used in many studies. Here, indicate whether each material, system or method listed is relevant to your study. If you are not sure if a list item applies to your research, read the appropriate section before selecting a response.

Materials & experimental systems

n/a	Involved in the study	
☒	☐	Antibodies
☒	☐	Eukaryotic cell lines
☒	☐	Palaeontology and archaeology
☒	☐	Animals and other organisms
☒	☐	Clinical data
☒	☐	Dual use research of concern

Methods

n/a	Involved in the study	
☒	☐	ChIP-seq
☒	☐	Flow cytometry
☒	☐	MRI-based neuroimaging
